# A spatial covariance ^123^I-5IA-85380 SPECT study of α4β2 nicotinic receptors in dementia with Lewy bodies

**DOI:** 10.1007/s00415-025-13332-5

**Published:** 2025-09-29

**Authors:** Judith R. Harrison, Sean J. Colloby, John T. O’Brien, John-Paul Taylor

**Affiliations:** 1 Translational and Clinical Research Institute, Faculty of Medical Sciences, Newcastle University, Campus for Ageing and Vitality, Newcastle Upon Tyne, NE4 5PL UK; 2 Department of Psychiatry, School of Clinical Medicine, University of Cambridge, Level E4, Box 189, Cambridge, CB2 0QC UK

**Keywords:** Dementia with Lewy bodies, Cholinergic, Acetylcholine, Nicotinic, Spatial covariance, SPECT

## Abstract

**Background:**

Cholinergic dysfunction, particularly involving nicotinic acetylcholine receptors (nAChRs), contributes to cognitive and psychiatric symptoms in dementia with Lewy bodies (DLB), yet spatial covariance patterns remain unexplored. We aimed to characterise these patterns using ^123^I-5-iodo-3-[2(S)-2-azetidinylmethoxy] pyridine (5IA-85380) SPECT (α4β2 nAChR assessment) and examine their association with cognitive function.

**Methods:**

Fifteen DLB and 16 healthy controls underwent ^123^5IA-85380 and rCBF ^99m^Tc-exametazime SPECT scanning. Voxel principal components analysis (PCA), generated PC images representing common intercorrelated voxels across subjects. Linear regression identified α4β2 nAChR and rCBF patterns distinguishing DLB from controls.

**Results:**

A distinct α4β2 nAChR pattern differentiated DLB from controls (F_1,29_ = 165.1, *p* < 0.001), that was dissimilar to rCBF changes. This pattern was characterised by decreased uptake in temporal pole, inferior frontal cortex, amygdala, olfactory cortex, insula, anterior/mid cingulate, and putamen, alongside preserved/increased uptake in sensorimotor cortex, fusiform and occipital lobe. These regions mapped onto default, salience, limbic, frontostriatal, sensorimotor and visual hubs. We then derived from patients, α4β2 nAChR patterns that correlated with CAMCOG_total_ (*r* =  – 0.52, *p* = 0.04), MMSE (*r* =  – 0.68, *p* = 0.01) and CAMCOG_memory_ (*r* =  – 0.70, *p* = 0.01), demonstrating a common topography of relative decreased binding in lateral/medial prefrontal, lateral temporal, fusiform, inferior parietal and thalamus along with relative preserved/increased binding in cingulate, insula, occipital and medial temporal regions: structures within a range of networks supporting executive, language, attention, motor and visual processing.

**Conclusion:**

These findings provide novel insights into the pathophysiology of DLB and may inform future therapeutic strategies targeting nAChRs.

## Introduction

Dementia with Lewy Bodies (DLB) is recognised as one of the most common forms of dementia, and is estimated to account for 15–20% of dementia cases in older people at autopsy, and second only to Alzheimer’s disease (AD) in prevalence [21, 33, 46]. DLB is distinguished clinically by a range of symptoms that include cognitive impairments, fluctuating attention, motor disturbances akin to parkinsonism, and prominent neuropsychiatric symptoms, most importantly visual hallucinations [34]. Despite its prevalence, the neurochemical mechanisms underlying its distinct cognitive and neuropsychiatric symptoms remain poorly understood.

Cholinergic neurotransmission is mediated by nicotinic and muscarinic acetylcholine receptors (nAChRs, mAChRs), which are integral to cognitive functions and their dysfunction is implicated in various neurodegenerative disorders [13, 45]. For nAChRs, the α4β2 subtype is among the most abundant and functionally significant variants in the human brain, with key roles in cognitive processing, neurotransmitter modulation, and reward pathways [15, 16]. Physiologically, they also play a fundamental role in working memory, sustained attention and learning by regulation of synaptic signalling within frontoparietal, hippocampal and thalamostriatal regions [29]. As such, α4β2 nAChRs are considered therapeutic targets, with α4β2 nAChR agonists assessed as potential treatments in AD and schizophrenia [17, 49]. Further, the cholinesterase inhibitor galantamine is an allosteric potentiator of α4β2 receptors [40], and has been posited to mediate some of its pro-cognitive effects [24]. In DLB, marked cholinergic dysfunction is a significant neurochemical feature and contributor to the cognitive, sleep and psychiatric symptoms [1], where the functional deficits appear to be more pronounced than in other neurodegenerative dementias, i.e., AD [25, 32]. In view of this and the apparent impact of α4β2 nAChRs and drugs such as galantamine on cognitive processes [9], detailed studies of these receptors and their relationship with cognitive signs in DLB are of interest.

Understanding how cholinergic receptors are altered at the systems network level may have implications for identifying specific symptom severity and potential treatment targets in DLB. Cholinesterase inhibitors (ChEIs) are the basis of symptomatic treatment in this condition [27], while there is also emerging evidence of focussing on M1/M4 mAChRs as a possible therapeutic [10]. Currently, there is varying response to ChEIs in DLB, with improvements only in about a third of patients in terms of cognition, although these agents appear to offer more consistent benefit in alleviating the behavioural and psychiatric symptoms [9, 31]. Therefore, examination at the network level could highlight important functionality that drive different aspects of the cholinergic system in DLB. One method of assessing such patterns is by multivariate approaches such as spatial covariance, a form of principal components analysis (PCA), which eliminates the notion of functional segregation and provides connectivity information between brain regions. In DLB, such methods have been implemented with glucose metabolism PET and perfusion SPECT imaging [23]. In fact, we successfully applied the technique to DLB patients scanned with ^123^I-iodo-quinuclidinyl-benzilate (QNB) SPECT, deriving a M1/M4 mAChR covariance disease pattern that characterised the receptor changes to cholinergic depletion, and identified cognitive and psychiatric symptom responder patterns following treatment with ChEIs [7].

Despite their functional significance in terms of cognitive processing and potential as a therapeutic target, little is known regarding how α4β2 nAChRs covary across brain regions in relation to cognitive severity in DLB. Although our previous study identified reduced α4β2 nAChR binding using 123I-5IA-85380 SPECT in frontal, striatal, temporal, and cingulate cortices in DLB [37], a network-level approach could yield new insights into the connectivity and functional organisation of these receptors in this condition. Subsequently, we applied spatial covariance to ^123^I-5IA-85380 SPECT scans, identifying intercorrelated patterns of α4β2 nAChRs that contrasted DLB from healthy controls. Next, by deriving patterns of α4β2 nAChRs that relate to specific cognitive measures, we aim to clarify how network level cholinergic disruptions contribute to the cognitive features in DLB. Lastly, since the assessment of ^123^I-5IA-85380 images alone is difficult due to potential confounding effects of perfusion and, to a lesser extent, cell loss, we studied for comparison the perfusion spatial covariance pattern in these individuals.

## Methods

### Subjects

The sample consisted of 31 non-smoking (> 10 years) subjects (15 DLB and 16 healthy elderly controls). Patients with DLB were recruited from a community-dwelling population following referral to local old age psychiatry services. Normal controls were recruited from friends and spouses of patients included in this and other research studies. Participants were recruited between 2002 and 2004. For DLB patients, the mean (± SD) duration of cognitive symptoms at time of scan was 2.9 ± 1.4 years, based on the clinical history and caregiver report. Exclusion criteria included major psychiatric illness, other neurodegenerative diseases (e.g., AD, frontotemporal dementia), significant vascular pathology on imaging, and contraindications for SPECT scanning. All subjects underwent both ^123^I-5IA-85380 and ^99m^Tc-exametazime SPECT scanning within a 3-month window per individual to minimise temporal variability. The study was approved by the Newcastle, North Tyneside and Northumberland local research ethics committee and the UK Department of Health’s Administration of Radioactive Substances Advisory Committee (ARSAC). All participants and/or nearest relative (for patients who lacked capacity) gave informed written consent.

### Assessments and diagnosis

Subjects underwent detailed physical, neurological and neuropsychiatric examinations, which included history, mental state and physical examination and, for DLB subjects, a standard blood screen with thyroid function tests, B12, folate and syphilis serology and CT brain scan. Cognitive function was evaluated with the mini-mental state examination (MMSE) and Cambridge Cognitive examination (CAMCOG) [39] tests. We also utilised the memory and executive subscales (CAMCOG_memory_, CAMCOG_exec_). Parkinsonism was assessed using part III (motor examination) of the unified Parkinson’s disease rating scale (UPDRS) [11]. Neuropsychiatric features and cognitive fluctuations were measured with the neuropsychiatric inventory (NPI) [8] and Clinician Assessment of Fluctuation scale [CAF; 48]. Diagnosis for all subjects was made by consensus between two experienced clinicians using the consensus criteria for probable DLB [35], while clinicopathological diagnosis was confirmed for 5 patients that subsequently died (4 DLB, 1 AD/DLB_mixed_). Controls had no signs or symptoms of cognitive disturbance, did not complain of poor memory and all scored within normal range of cognitive tests. Four DLB patients were being treated with cholinesterase inhibitors (donepezil) for at least 12 weeks at the time of their ^123^I-5IA-85380 scan, while the remaining 11 patients had never received cholinesterase medication.

### Radiochemistry

Radiosynthesis of ^123^I-5IA-85380 was produced from the corresponding stanyl precursor, 5-SnBu_3_-A85380, by electrophilic iododestanylation, and performed according to details previously described (O’Brien et al. 2007).

### Acquisition

Subjects were scanned with a triple-headed rotating gamma camera (Picker 3000XP), 2 h post injection of 185 MBq of ^123^I-5IA-85380 using an earlier reported imaging protocol (O’Brien et al. 2007). Within three months of ^123^I-5IA-85380 scanning, all subjects underwent ^99m^Tc-exametazime regional cerebral blood flow (rCBF) SPECT in accordance with methods described previously [6].

### Spatial pre-processing

All SPECT scans were spatially normalised to match, as appropriate, a ^123^I-5IA-85380 or ^99m^Tc-exametazime SPECT template in standard stereotactic MNI space using linear image registration software (FLIRT: https://fsl.fmrib.ox.ac.uk/fsl/docs/#/registration/flirt/index). Generation of the template images have been described [6, 36]. The spatially transformed images were then smoothed with a 10 mm FWHM 3D Gaussian filter.

### Spatial covariance analysis

Spatial covariance was simultaneously applied to ‘n’ pre-processed (registered and smoothed) ^123^I-5IA-85380 SPECT scans using covariance software (http://www.nitrc.org/projects/gcva_pca/) [18], capturing the major sources of variation, producing (n – 1) principal component (PC) images organised in a descending order of decreasing variance. A mask image defined the brain volume subspace for voxel analyses. Global means (within brain mask) for each subject were computed and subtracted from the data matrix to ensure the PC images were not influenced by individual differences in global tracer uptake. For each PC image, voxels had positive and negative weights representing the sign and strength of voxel covariance, that remained fixed across subjects. Specifically, positive and negative voxels were interpreted as concomitant normalised increased and decreased α4β2 nicotinic binding, respectively. The degree to which a subject expressed a PC image (PC_1_, PC_2_,……., PC_n-1_) was by means of the subject-scaling factor (SSF_1_, SSF_2_,…, SSF_n-1_), formed from effectively the ‘dot product’ (PC_i_ ∙ SPECT_α4β2_) of two images, yielding a scalar value (SSF_i_) representing the projection of PC_i_ onto an individual’s nicotinic SPECT scan. Therefore, a high SSF_i_ score for the corresponding PC_i_ image, indicates a greater normalised increased binding in voxels with positive weights and a greater normalised decreased binding in voxels with negative weights.

To derive the nicotinic spatial covariance pattern (SCP_α4β2_) that discriminated DLB from controls; all subject scale factor scores (SSF_1_, SSF_2_,…, SSF_n-1_) were entered into a linear regression model as independent variables with ‘group’ as the dependent measure. Akaike’s information criteria (AIC) determined how many SSFs (PCs), should be included in the regression model to achieve optimum trade-off between goodness of fit and model simplicity [4]. The set of SSFs (PCs) generating the lowest AIC value were chosen as predictors for the model, where the resulting linear combination formed the composite pattern (SCP_α4β2_). The extent to which each subject expressed the SCP_α4β2_ was by the SSF_α4β2_, i.e., SSF_α4β2_ = SCP_α4β2_ ∙ SPECT_α4β2_ of two scans, representing the projection of the SCP_α4β2_ onto each individual’s nicotinic SPECT image. Spatial covariance was then applied to the perfusion scans, primarily to assess whether the α4β2 nicotinic disease pattern (SCP_α4β2_) differed from perfusion. Therefore, positive and negative weights of these images were interpreted as concomitant relative/normalised increased and decreased rCBF/perfusion respectively. The analysis generated the SCP_rCBF_ that separated DLB from controls, with subject expression scores (SSF_rCBF_).

We then investigated α4β2 nicotinic SCPs that correlated with various cognitive measures in DLB (CAMCOG_total_, MMSE, CAMCOG_exec_, CAMCOG_memory_, CAF). This involved conducting separate covariance analyses, generating a set of DLB specific PCs, expressed by the SSFs, which were entered into a regression model as predictors, with either CAMCOG_total_, MMSE, CAMCOG_exec_, CAMCOG_memory_ and CAF as the dependent. The resulting linear combinations, with lowest AIC, defined the α4β2 nicotinic ‘cognitive’ SCPs, with their specific individual pattern expression scores (SSF).

Stability and reliability of the SCPs were assessed by bootstrap resampling (1000 iterations), to identify areas that contributed to the patterns with high confidence. This transforms the voxel weights of each SCP into Z maps, computed as the ratio of voxel weight and bootstrap standard deviation. The Z-statistic follows roughly a standard normal distribution where a one-tailed p ≤ 0.05 infers a threshold of |Z|≥ 1.64 [19]. Anatomical labelling of the Z maps were performed using the image visualisation software ‘FSLeyes’ (https://fsl.fmrib.ox.ac.uk/fsl/docs/#/utilities/fsleyes), which contains various anatomical brain atlases from which the labels were reported.

### Statistical analyses

Analysis used IBM SPSS v. 25.0.0.1 and R (v. 4.0.3, https://www.R-project.org/). Variables were tested for normality and variance homogeneity using Shapiro–Wilk and Levene’s tests, respectively. The data was examined using parametric (ANOVA F, Welch’s ANOVA W) and non-parametric (Mann–Whitney *U*, χ^2^) tests. Correlations were assessed with Pearson coefficients (*r*), with Benjamini–Hochberg multiple comparisons correction (*p*´) applied across all cognitive measures.

## Results

### Subject demographics and clinical characteristics

Table 1 shows demographic and clinical characteristics of study participants. Groups were similarly matched for age and gender, while as expected, cognitive and clinical variables differed (*p* < 0.001).

**Table 1:** Demographic, clinical and neuropsychological data for individuals studied with ^123^I-5IA-85380 SPECT

	Control	DLB	Statistic, *p*-value
*n*	16	15	
Sex (m:f)	10:6	12:3	χ^2^_(1)_ = 1.1, *p* = 0.3
Age	75.4 ± 4.5	74.0 ± 6.7	F_1,29_ = 0.5, *p* = 0.5
MMSE	28.8 ± 1.1	21.0 ± 6.6	**F**_**1,29**_** = 23.1, *p* < 0.001**
CAMCOG	97.8 ± 3.8	74.2 ± 16.5	**F**_**1,27**_** = 33.5, *p* < 0.001**
CAMCOG_memory_	24.0 ± 1.8	17.4 ± 3.9	**F**_**1,27**_** = 36.1, *p* < 0.001**
CAMCOG_exec_	23.3 ± 3.1	13.4 ± 6.2	**F**_**1,27**_** = 31.0, *p* < 0.001**
UPDRS III	1.6 ± 1.6	32.9 ± 12.8	**U = 239.0, Z = 4.7, *p* < 0.001**
CAF	n/a	8.4 ± 3.4	
NPI	n/a	13.2 ± 11.7	
Medications			
ChEIs (yes)	n/a	4	

Data expressed as mean ± 1 SD

*DLB*  Dementia with Lewy bodies, *MMSE* Mini-Mental State Examination, *CAMCOG* Cambridge Cognitive Examination, *CAMCOG*_*memory*_ memory component of CAMCOG, *CAMCOG*_*exec*_ executive function component of CAMCOG, *UPDRS III* Unified Parkinson’s disease related scale (motor subsection), *CAF* Cognitive fluctuations score, *NPI* Neuropsychiatric Inventory, *n/a* not applicable, *ChEIs* cholinesterase inhibitors

**Bold** text denotes significant differences

### Spatial covariance

**Fig. 1:**
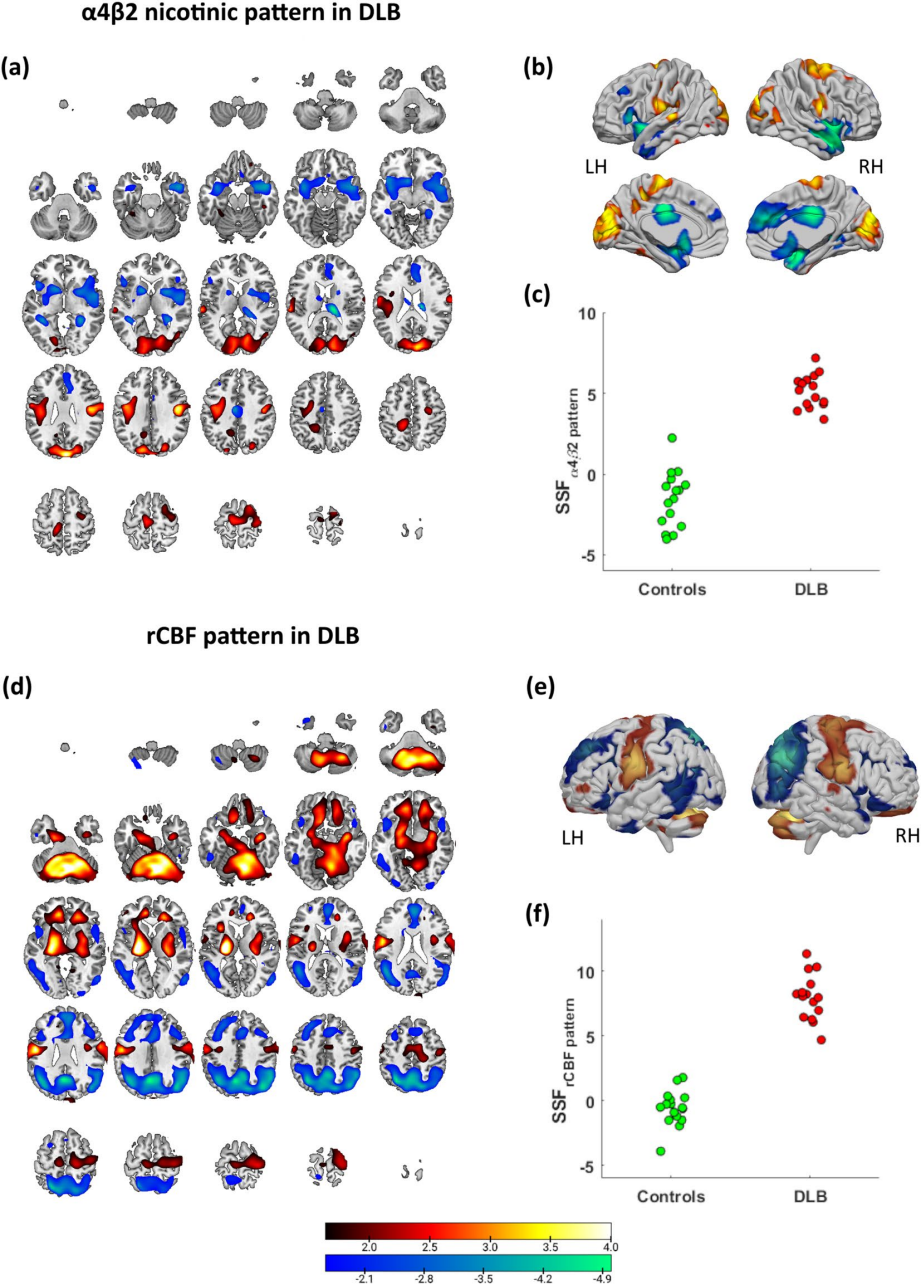
α4β2 nicotinic disease related pattern in DLB projected onto axial (a) and cortical surface rendered MRI brain template (**b**), with scatter plot of subject scores (SSF_α4β2 pattern_) vs. group (**c**). Corresponding rCBF disease related pattern in DLB projected onto axial (**d**) and rendered MRI brain template (**e**), with scatter plot of subject scores (SSF_rCBF pattern_) vs. group (**f**). Images displayed neurologically (left is left)

The α4β2 nAChR SCP, a composite image of PC_1_, PC_3_, PC_5_, PC_6_, PC_7_ and PC_9_, distinguished DLB from controls (Fig. 1a, b). SSF_α4β2_ scores, representing the extent to which subjects expressed the topography, was higher in DLB than controls (mean ± SD; DLB = 5.1 ± 1.1, controls =  – 1.6 ± 1.7; F_1,29_ = 165.1, *p* < 0.001; Fig. 1c). The pattern was characterised by relative decreased binding (blue) in temporal pole, inferior frontal, superior medial frontal, amygdala, thalamus, olfactory cortex, insula, anterior/mid cingulate and putamen, with relative preserved/increased uptake (red) in sensorimotor, fusiform and structures within the occipital lobe. The decreased nAChR pattern implicated regions that lie within the default, salience, limbic and corticostriatal networks, while the preserved/increased nAChR pattern involved regions within sensorimotor and visual hubs. Table 2 depicts details of specific regions contributing to the α4β2 nAChR disease related pattern in DLB with high confidence (|Z|≥ 1.64, *p* ≤ 0.05).

**Table 2:** Regions contributing to the nicotinic α4β2 disease related pattern in DLB with high confidence (|Z|≥ 1.64, *p* ≤ 0.05)

Hemisphere	MNI coordinates	Region	Z score
R	49, 2, – 31	Inferior temporal gyrus	– 2.1
R	48, 4, – 23	Middle temporal gyrus	– 2.5
R	38, 5, – 21	Temporal pole	– 2.4
L	– 33, 5, – 21	Temporal pole	– 2.1
R	32, 4, – 20	Amygdala	– 2.4
L	– 30, 3, – 20	Amygdala	– 2.0
R	25, 12, – 18	Olfactory cortex	– 1.8
L	– 14, 11, – 18	Olfactory cortex	– 1.7
R	5, 24, – 16	Rectal gyrus	– 1.8
L	– 32, – 12, – 16	Hippocampus	– 1.9
L	– 42, 10, – 4	Insula	– 2.0
R	40, 16, – 4	Insula	– 2.1
R	54, – 3, – 2	Superior temporal gyrus	– 2.3
L	– 26, 5, – 2	Putamen	– 1.9
R	26, 7, – 2	Putamen	– 2.3
R	47, 27, 4	Inferior frontal gyrus	– 1.8
L	47, 23, 4	Inferior frontal gyrus	– 2.1
R	4, 53, 21	Superior medial frontal gyrus	– 1.9
L	0, 43, 27	Superior medial frontal gyrus	– 1.8
R	12, – 26, 12	Posterior thalamus	– 2.3
R	5, 35, 18	Anterior cingulate	– 1.9
R	7, 34, 31	Mid cingulate	– 1.8
L	– 5, – 11, 36	Mid cingulate	– 2.2
L	−37, −37, – 24	Fusiform	1.8
R	43, – 32, – 24	Fusiform	2.0
L	– 8, – 20, 73	Paracentral lobule	2.1
R	7, −20, 73	Paracentral lobule	2.0
L	– 43, – 7, 40	Precentral gyrus	2.2
R	27, – 24, 73	Precentral gyrus	2.0
L	– 45, – 10, 40	Postcentral gyrus	2.4
R	49, – 10, 40	Postcentral gyrus	2.8
R	23, 4, 67	Superior frontal gyrus	2.0
L	– 17, – 82, 26	Superior occipital gyrus	2.1
R	26, – 82, 26	Superior occipital gyrus	2.1
L	– 4, – 88, 26	Cuneus	2.5
R	12, – 87, 26	Cuneus	2.6
L	– 19, – 87, 20	Middle occipital gyrus	1.8
R	30, – 88, 20	Middle occipital gyrus	1.9
L	– 7, – 81, 14	Calcarine	2.2
R	14, – 79, 14	Calcarine	2.3
L	– 7, – 78, 4	Lingual gyrus	2.0
R	16, – 79, 2	Lingual gyrus	1.9

The corresponding regional cerebral blood flow SCP, a composite image of PC_1_, PC_2_, PC_3_, PC_4_, PC_5_, PC_10_ and PC_12_, also fully differentiated DLB from controls (Fig. 1d and e), where SSF_rCBF_ scores differed between groups (DLB = 8.0 ± 1.8, controls =  – 0.6 ± 1.4; F_1,29_ = 230.1, *p* < 0.001; Fig. 1f). The pattern mainly comprised of relative decreased rCBF (blue) in inferior/middle temporal, inferior occipital, cuneus/precuneus, inferior/superior parietal, medial frontal and anterior cingulate areas with relative increased rCBF (red) in cerebellum, lentiform nucleus, pre/post central, thalamus, olfactory cortex and orbitofrontal regions bilaterally. Table 3 details the specific regions that significantly contributed to the rCBF disease related pattern in DLB (|Z|≥ 1.64, *p* ≤ 0.05).

**Table 3:** Regions contributing to the corresponding rCBF pattern in DLB with high confidence (|Z|≥ 1.64, *p* ≤ 0.05)

Hemisphere	MNI coordinates	Region	Z score
R	49, 17, – 14	Temporal pole	– 1.8
L	– 44, 19, – 14	Temporal pole	– 2.0
R	53, – 73, −5	Inferior temporal gyrus	– 2.2
L	– 55, – 50, – 14	Inferior temporal gyrus	– 2.1
R	53, – 76, 2	Middle temporal gyrus	– 2.5
L	– 60, – 47, – 5	Middle temporal gyrus	– 2.1
R	31, – 95, −5	Inferior occipital gyrus	– 2.1
L	– 35, – 86,−5	Inferior occipital gyrus	– 2.0
R	10, – 76, 32	Cuneus	– 3.1
L	– 7, – 76, 32	Cuneus	– 3.3
R	4, 49, 11	Anterior cingulate	– 2.3
L	– 1, 39, 18	Anterior cingulate	– 2.3
R	10, 56, 18	Superior medial frontal gyrus	– 2.4
L	– 2, 53, 18	Superior medial frontal gyrus	– 2.6
R	38, 27, 32	Middle frontal gyrus	– 2.3
L	– 30, 36, 32	Middle frontal gyrus	– 2.1
R	44, 21, 26	Inferior frontal gyrus	– 2.0
L	– 46, 17, 26	Inferior frontal gyrus	– 2.0
R	5, – 59, 26	Precuneus	– 3.5
L	– 4, – 59, 26	Precuneus	– 4.0
R	58, – 52, 26	Inferior parietal	– 3.0
L	– 47, – 50, 26	Inferior parietal	– 3.1
R	13, – 69, 51	Superior parietal	– 3.6
L	– 21, – 69, 51	Superior parietal	– 3.5
R	7, 23, 51	Supplementary motor area	– 2.2
L	– 1, 22, 51	Supplementary motor area	– 2.0
L	– 18, – 60, – 32	Cerebellum	4.0
R	22, – 63, – 32	Cerebellum	4.6
L	– 24, – 7, – 21	Hippocampus	2.7
R	36, – 12, – 21	Hippocampus	3.5
L	– 16, 13, – 15	Olfactory cortex	2.8
R	24, 13, – 15	Olfactory cortex	2.1
L	– 8, 24, – 15	Gyrus rectus	3.5
R	21, 17, – 15	Gyrus rectus	2.2
L	– 18, 28, – 15	Orbitofrontal cortex	2.6
R	20, 28, – 15	Orbitofrontal cortex	2.7
L	– 24, 7, −2	Putamen	3.0
R	27, 7, – 2	Putamen	2.6
L	– 20, – 3, – 2	Globus pallidus	4.4
R	23, – 3, – 2	Globus pallidus	2.2
L	– 14, – 16, 6	Thalamus	3.9
R	18, – 14, 6	Thalamus	2.3
L	– 53, – 5, 19	Postcentral gyrus	3.9
R	65, – 5, 19	Postcentral gyrus	2.5
L	– 57, – 4, 34	Precentral gyrus	3.4
R	44, – 6, 34	Precentral gyrus	2.1

**Fig. 2:**
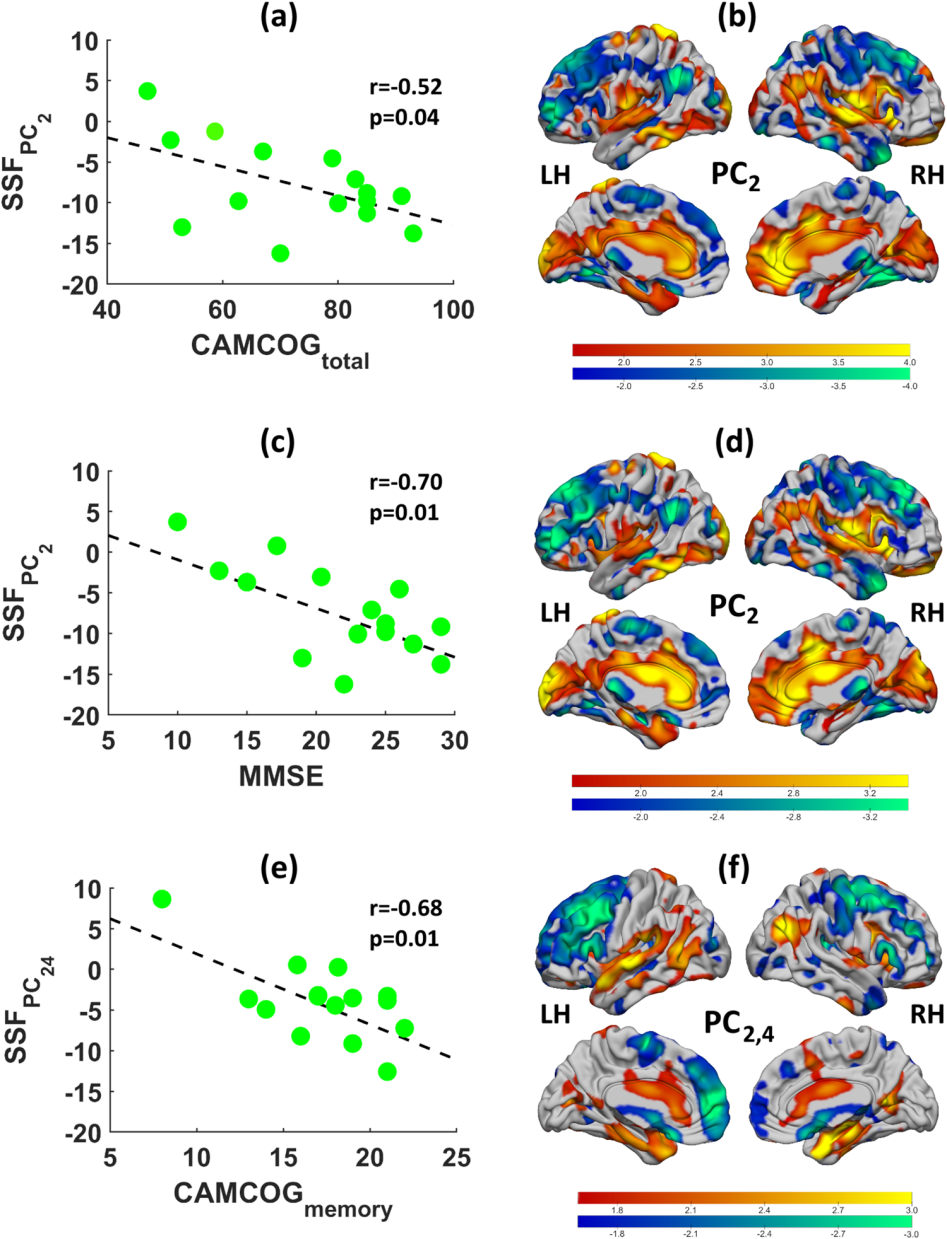
Cognitive (CAMCOG_total_ (**b**), MMSE (**d**) and CAMCOG_memory_ (**f**)) α4β2 nicotinic patterns in DLB projected onto cortical surface rendered MRI brain templates. Scatter plots depicting the relationship between specific pattern expression scores (SSF) with CAMCOG_total_ (**a**), MMSE (**c**) and CAMCOG_memory_ (**e**)

In DLB, we then derived a series of ‘cognitive’ α4β2 nicotinic spatial covariance patterns that individually correlated with CAMCOG_total_ (PC_2_; *r* =  – 0.52, *p*´ = 0.04), MMSE (PC_2_; *r* =  – 0.68, *p*´ = 0.01), CAMCOG_exec_ (PC_2_; *r* =  – 0.45, *p*´ = 0.06), CAMCOG_memory_ (PC_2,4_; *r* =  – 0.70, *p*´ = 0.01) and CAF (PC_5_; *r* =  – 0.55, *p*´ = 0.04). However, CAMCOG_exec_ was not significant, while the topography associated with CAF, post bootstrap resampling, revealed no significant regions that contributed to the pattern (|Z|< 1.64, *p* > 0.05). The α4β2 nAChR patterns associated with CAMCOG_total,_ MMSE and CAMCOG_memory_ in DLB are illustrated in Fig. 2b, d and f along with plots of the related pattern expressions as a function of these measures respectively (Fig. 2a,c,e). Tables 4, 5 and 6 outline regions contributing, correspondingly, to the CAMCOG_total,_ MMSE and CAMCOG_memory_ patterns. These variables appeared to converge onto a common topography, i.e., PC_2_, and consisted of relative decreased α4β2 nicotinic uptake (cold colours) in medial/inferior/middle/superior frontal, fusiform, inferior/middle temporal, inferior parietal, thalamus and precentral/supplementary motor areas, and relative preserved/increased α4β2 nicotinic uptake (warm colours) in anterior/posterior cingulate, insula, precuneus, cuneus/calcarine and hippocampus/parahippocampal regions. The DLB ‘cognitive impairment’ pattern with decreased nAChR binding appears to be involved in areas within default, frontoparietal, ventral visual and sensorimotor networks, while the increased/preserved nAChR binding implicated regions within salience, primary visual and limbic circuits.

**Table 4:** Regions contributing to the DLB ‘cognitive (CAMCOG_total_)’ α4β2 nicotinic pattern with high confidence (|Z|≥ 1.64, *p* ≤ 0.05)

Hemisphere	MNI coordinates	Region	Z score
L	– 19, – 68, – 40	Cerebellum	– 4.4
R	24, – 68, – 40	Cerebellum	– 4.2
L	– 51, – 25, – 18	Inferior temporal gyrus	– 4.2
R	57, – 35, – 18	Inferior temporal gyrus	– 4.2
L	– 45, – 2, – 26	Middle temporal gyrus	– 5.1
R	55, 5, – 26	Middle temporal gyrus	– 2.6
L	– 34, – 41, – 20	Fusiform	– 2.1
R	34, – 41, – 21	Fusiform	– 1.8
L	– 16, – 18, 1	Thalamus	– 2.5
R	16, – 18, 1	Thalamus	– 2.5
L	– 36, – 87, 7	Middle occipital gyrus	– 2.4
R	40, – 80, 7	Middle occipital gyrus	– 5.1
L	– 17, 15, – 20	Inferior frontal gyrus	– 2.2
R	24, 17, – 20	Inferior frontal gyrus	– 2.3
L	– 8, 18, – 19	Rectal gyrus	– 2.5
R	6, 19, – 19	Rectal gyrus	– 2.3
L	– 9, 35, 48	Medial frontal gyrus	– 4.4
R	11, 37, 48	Medial frontal gyrus	– 4.3
L	– 44, 29, 34	Middle frontal gyrus	– 4.3
R	31, 41, 34	Middle frontal gyrus	– 3.7
L	– 21, 41, 41	Superior frontal gyrus	– 3.6
R	25, 43, 41	Superior frontal gyrus	– 3.2
L	– 47, – 1, 48	Precentral gyrus	– 3.6
R	50, 3, 48	Precentral gyrus	– 4.0
L	– 51, – 53, 37	Inferior parietal	– 4.4
R	40, – 46, 41	Inferior parietal	– 2.2
L	– 8, – 12, 64	Supplementary motor area	– 3.4
R	6, – 19, 64	Supplementary motor area	– 3.3
L	– 6, 17, 30	Anterior cingulate	2.9
R	7, 14, 30	Anterior cingulate	2.6
L	– 6, – 45, 31	Posterior cingulate	2.0
R	12, – 44, 31	Posterior cingulate	2.2
L	– 30, – 25, – 10	Hippocampus	4.0
R	29, – 25, – 10	Hippocampus	4.0
L	– 19, – 26, – 15	Parahippocampal gyrus	2.2
R	34, – 23, – 15	Parahippocampal gyrus	3.6
L	– 55, – 2, – 2	Superior temporal gyrus	3.0
R	60, – 5, – 2	Superior temporal gyrus	3.6
L	– 41, 8, – 2	Insula	2.2
R	38, 17, – 2	Insula	4.5
L	– 3, – 85, 23	Cuneus	2.6
R	9, – 84, 23	Cuneus	2.0
L	– 10, – 56, 23	Precuneus	2.3
R	17, – 56, 23	Precuneus	3.6
L	– 14, – 68, 15	Calcarine	1.8
R	12, – 74, 15	Calcarine	2.1

**Table 5:** Regions contributing to the DLB ‘cognitive (MMSE)’ α4β2 nicotinic pattern with high confidence (|Z|≥ 1.64, *p* ≤ 0.05)

Hemisphere	MNI coordinates	Region	Z score
L	– 19, – 68, – 40	Cerebellum	– 4.4
R	24, – 68, – 40	Cerebellum	– 4.2
L	– 51, – 25, – 18	Inferior temporal gyrus	– 4.2
R	57, – 35, – 18	Inferior temporal gyrus	– 4.3
L	– 45, – 2, – 26	Middle temporal gyrus	– 5.1
R	55, 5, – 26	Middle temporal gyrus	– 2.7
L	– 34, – 41, – 20	Fusiform	– 2.3
R	39, – 45, −21	Fusiform	– 2.0
L	– 17, 15, – 20	Orbitofrontal cortex	– 2.1
R	24, 17, – 20	Orbitofrontal cortex	– 2.3
L	– 8, 18, – 19	Rectal gyrus	– 2.5
R	6, 19, – 19	Rectal gyrus	– 2.2
L	– 16, – 18, 1	Thalamus	– 2.5
R	16, – 18, 1	Thalamus	– 2.5
L	– 36, – 87, 7	Middle occipital gyrus	– 2.4
R	40, – 80, 7	Middle occipital gyrus	– 5.1
L	– 9, 35, 48	Medial frontal gyrus	– 4.4
R	11, 37, 48	Medial frontal gyrus	– 4.3
L	– 44, 29, 34	Middle frontal gyrus	– 4.0
R	31, 41, 34	Middle frontal gyrus	– 4.0
L	– 21, 41, 41	Superior frontal gyrus	– 3.2
R	25, 43, 41	Superior frontal gyrus	– 2.6
L	– 47, – 1, 48	Precentral gyrus	– 3.6
R	50, 3, 48	Precentral gyrus	– 4.2
L	– 51, – 53, 37	Inferior parietal	– 4.4
R	40, – 46, 41	Inferior parietal	– 2.1
L	– 8, – 12, 64	Supplementary motor area	– 3.4
R	6, – 19, 64	Supplementary motor area	– 3.7
L	– 6, 17, 30	Anterior cingulate	2.9
R	7, 14, 30	Anterior cingulate	2.6
L	– 6, – 45, 31	Posterior cingulate	1.9
R	12, – 44, 31	Posterior cingulate	2.5
L	– 30, – 25, – 10	Hippocampus	4.0
R	29, – 25, – 10	Hippocampus	4.0
L	– 23, – 26, – 16	Parahippocampal gyrus	3.5
R	34, – 26, – 16	Parahippocampal gyrus	2.8
L	– 51, – 2, – 2	Superior temporal gyrus	2.0
R	60, – 5, – 2	Superior temporal gyrus	2.2
L	– 41, 8, – 2	Insula	2.1
R	38, 17, – 2	Insula	4.5
L	– 3, – 85, 23	Cuneus	2.6
R	9, – 84, 23	Cuneus	2.0
L	– 10, – 56, 23	Precuneus	2.3
R	17, −56, 23	Precuneus	3.6
L	– 14, – 68, 15	Calcarine	1.9
R	12, – 74, 15	Calcarine	2.1

**Table 6:** Regions contributing to the DLB ‘cognitive (CAMCOG_memory_)’ α4β2 nicotinic pattern with high confidence (|Z|≥ 1.64, *p* ≤ 0.05)

Hemisphere	MNI coordinates	Region	Z score
L	– 45, – 6, – 36	Inferior temporal gyrus	– 2.1
R	47, 6, – 36	Inferior temporal gyrus	– 2.2
L	– 55, – 35, – 2	Middle temporal gyrus	– 2.2
R	53, – 44, – 2	Middle temporal gyrus	– 2.4
L	– 11, 53, – 8	Orbitofrontal cortex	– 2.7
R	28, 51, – 8	Orbitofrontal cortex	– 4.4
L	– 13, 10, – 16	Olfactory cortex	– 2.8
R	14, 11, – 16	Olfactory cortex	– 3.0
L	– 7, 22, – 18	Rectal gyrus	– 2.6
R	2, 21, – 18	Rectal gyrus	– 3.4
L	– 14, – 22, 7	Thalamus	– 3.3
R	12, – 23, 7	Thalamus	– 3.1
L	– 35, – 87, 9	Middle occipital gyrus	– 2.1
R	42, – 77, 9	Middle occipital gyrus	– 2.9
L	– 49, 28, 9	Ventrolateral prefrontal cortex	– 2.3
R	56, 27, 9	Ventrolateral prefrontal cortex	– 4.1
L	– 6, 42, 38	Medial frontal gyrus	– 2.5
R	9, 31, 42	Medial frontal gyrus	– 2.0
L	– 36, – 41, 22	Middle frontal gyrus	– 3.2
R	31, 42, 22	Middle frontal gyrus	– 3.6
L	– 15, 32, 48	Superior frontal gyrus	– 3.3
R	21, 29, 48	Superior frontal gyrus	– 3.5
L	– 40, 2, 48	Precentral gyrus	– 3.4
R	44, 1, 48	Precentral gyrus	– 3.1
L	– 11, 7, 61	Supplementary motor area	– 3.2
L	– 21, – 6, – 22	Hippocampus	2.2
R	32, – 4, – 22	Hippocampus	4.4
L	– 18, – 23, – 19	Parahippocampal gyrus	2.3
R	23, – 23, – 19	Parahippocampal gyrus	4.4
L	– 58, – 32, 24	Superior temporal gyrus	2.9
R	53, – 48, 24	Superior temporal gyrus	2.5
L	– 37, 9, 5	Insula	1.9
R	37, 5, 5	Insula	3.1
L	– 10, – 77, 22	Cuneus	2.4
R	15, – 60, 22	Cuneus	3.1
L	– 4, – 60, 9	Calcarine	2.8
R	15, – 60, 9	Calcarine	3.6

## Discussion

We applied spatial covariance analysis to ^123^I-5IA-85380 SPECT scans, an α4β2 nAChR ligand, to investigate nicotinic cholinergic function in DLB. We derived disease and cognitive related α4β2 nicotinic covariance patterns, which implied the presence of several dysfunctional cholinergic networks in this condition. The disease pattern showed decreased nAChRs within default, salience, limbic and frontostriatal networks along with preserved/increased nAChRs within sensorimotor and visual hubs. The ‘cognitive impairment’ pattern demonstrated decreased nAChRs within default, frontoparietal, ventral visual and sensorimotor networks as well as preserved/increased nAChRs within salience, primary visual and limbic hubs. These findings reveal novel insights into the network-level organisation of nAChR availability in DLB, extending beyond our previous region-based analyses [37]. The nAChR patterns in DLB appear to affect hubs supporting executive, language, attention and sensory functions. In addition, compared to our prior assessment of M1/M4 mAChRs in DLB [7], a more diffuse pattern was observed with α4β2 nAChRs, suggesting these receptor subtypes are perhaps more susceptible than M1/M4 mAChRs in this condition.

A nicotinic covariance pattern was identified that contrasted DLB from controls and consisted of concomitant decreased and preserved/increased uptake in several brain regions. The decreased pattern comprised of areas within temporal pole, inferior frontal, superior medial frontal, amygdala, thalamus, olfactory cortex, insula, anterior/mid cingulate and putamen, along with a preserved/increased pattern within sensorimotor, fusiform and occipital structures. The disease related pattern incorporated several potential brain networks important to DLB symptomatology, i.e., frontostriatal, motor and ventral visual stream [3, 22]. As frontostriatal circuits support executive, attention and cognitive flexibility [5], decreased uptake within these regions may affect the executive/attentional deficits. The preserved/increased uptake within sensorimotor, fusiform and occipital lobe, whilst part of a possible compensatory response to the loss of acetylcholine; the persistent upregulation of nAChRs could also lead to neuronal and subsequent network instability [28], which may contribute to some of the motor and visuospatial abnormalities. Compared with our previous covariance assessment of M1/M4 mAChRs in DLB [7], where a more discreet disease related topography was found; the pattern of preserved or upregulated occipital binding observed with both M1/M4 mAChR and α4β2 nAChR ligands could indicate a mutual role in the pathophysiology of visuospatial dysfunction. The integrity of the cholinergic system in DLB, albeit in small groups, has also been examined with PET and SPECT radioligands targeting the vesicular acetylcholine transporter (VAChT). Specifically, ^18^F-fluoroethoxybenzovesamicol PET imaging showed decreased VAChT binding, describing a topography of cholinergic vulnerability in key neural hubs involving cingulo-opercular, salience, visual attention, corticostriatal and spatial navigation [26]. Similarly, ^123^I-iodobenzovesamicol SPECT imaging identified regions of reduced uptake within cingulo-opercular, salience, frontoparietal, dorsal attention and corticostriatal circuits [32]. These findings indicate that multiple aspects of the cholinergic system are subject to significant patho-modulation in DLB, with consistent involvement of the corticostriatal, salience and cingulo-opercular networks.

We derived a series of α4β2 nAChR patterns that were individually associated with CAMCOG_total_, MMSE and CAMCOG_memory_. As expected, these variables converged onto a common topography of cognitive impairment, which consisted of relative decreased nAChR binding in frontal, temporal, fusiform, inferior parietal, thalamus and motor cortices, and relative preserved/increased nAChR uptake in cingulate, insula, precuneus, cuneus/calcarine and hippocampus/parahippocampus. The DLB nicotinic ‘cognitive impairment’ pattern of decreased binding implicated regions within default, frontoparietal, ventral visual and sensorimotor networks, along with preserved/increased binding within salience, primary visual and limbic circuits. The pattern of reduced α4β2 nAChRs encompassed hubs associated with memory retrieval, cognitive slowing, executive and attentional function, as well as motor and sensory processing. This aligned with a previous study conducted across the REM sleep behaviour disorder (RBD) and Lewy body disease (LBD) spectrum, including individuals with PD and DLB, of basal forebrain atrophy, a structural marker of cholinergic degeneration, that showed significant correlations with attentional, executive and memory deficits [50]. The preserved/increased α4β2 nAChR signal observed could be adaptive mechanisms of specific networks, potentially in response to the marked cognitive and cholinergic changes. Interestingly, our previous study demonstrated relative preservation of M1/M4 mAChRs within attention/executive, ventral visual and salience hubs as a possible criteria for cognitive responsiveness to cholinesterase inhibitor therapy in DLB [7]. These and the present findings reinforce the notion that regions associated with cholinergic attention, executive, ventral visual and salience networks appear central to the pathophysiology of cognition in DLB, and therefore represent promising treatment targets with mAChR or nAChR agonists. Galantamine, may also have specific impacts on nicotinic α4β2 networks that we have evidenced in the present study. In an open label trial in DLB, this agent appeared to improve cognitive fluctuations and psychiatric symptoms, whereas for cognition the picture was less clear [43]. However, such results are tentative and require evidence from randomised controlled trials to establish its efficacy in DLB.

The corresponding rCBF pattern, dissimilar from the nAChR topography, comprised of relative decreased perfusion in temporal, occipital, parietal, medial frontal and anterior cingulate areas with relative increased perfusion in cerebellum, lentiform nucleus, central, thalamus, olfactory and orbitofrontal regions; a pattern similar to that observed in our previous rCBF assessment of DLB (in an independent cohort) [7]. Others using spatial covariance approaches with FDG-PET have identified changes in occipitoparietal (hypometabolism) and basal ganglia, limbic as well as motor cortices (hypermetabolism) in DLB as compared to controls [44], that chimes with the present rCBF findings. Also, regions of relative reduction in rCBF appeared to mainly involve areas within default, dorsal attention, visual and frontoparietal hubs, and follows a consistent topography of reviewed structural and functional imaging studies in DLB and Lewy body disease (LBD) of disruptions to default, frontoparietal and sensorimotor networks [2, 20, 30, 47]. However, our earlier fMRI analysis of DLB showed alterations to frontoparietal [38] rather than default networks [38, 41]. Although such studies were in mild disease whereas in this study, patients were at a more advanced stage of illness. Frontoparietal modulation appears to characterise early-stage DLB, whereas alterations to default mode hubs, commonly observed in AD, may emerge later as AD co-pathology becomes more prominent [12]. Regions of relative increased rCBF predominantly involve cerebellar, sensorimotor, limbic and basal ganglia–thalamocortical circuits. Several of these areas have been reported to show elevated activity within resting-state networks in DLB, including increased cerebellar–sensorimotor connectivity [42], and strengthened precuneal–putamen coupling [14]. However, it is unclear whether these increases are purely compensatory responses to the marked functional changes seen in this group [48].

Interpretation of preserved or increased α4β2 binding requires caution. Such patterns may reflect compensatory upregulation of nAChRs in spared regions, disease heterogeneity, or technical influences including intensity normalization and partial volume effects due to atrophy. We attempted to mitigate the latter via standard preprocessing and bootstrapping, though residual effects may persist. Further studies using partial volume correction or kinetic modeling would help disambiguate these contributions. An advantage of this study was the majority of DLB patients were not receiving ChEI treatment, therefore minimising the potential confound of reduced ^123^I-5IA-85380 binding resulting from increased competition between elevated endogenous acetylcholine and the radioligand. Another strength was the availability of both nicotinic and perfusion SPECT scans for all participants. Although the study sample was modest, the robustness of our findings was supported by stratified bootstrap resampling (1000 iterations), which yielded consistent spatial patterns with high voxel-wise confidence. In addition, we have previously applied spatial covariance to muscarinic receptor and metabolic imaging in DLB, identifying replicable network-level signatures using similar methods [7]. Nonetheless, independent validation in larger and more diverse cohorts is required. In addition, there was a minority of autopsy confirmed diagnoses. Since the patterns were largely separate, we made no attempt to establish whether the observed cholinergic patterns in DLB were attributed exclusively to alterations in α4β2 nAChRs or to some degree by the rCBF changes. Such a correction would have involved either intensity normalisation of the scans to a brain region devoid of specific receptors, assuming non-specific binding is similarly affected by rCBF, or using kinetic modelling with an arterial input function. However, selection of an appropriate non-specific reference region was uncertain, and arterial input function measurements were not obtained during scanning for either patient or control groups. Nonetheless, both ^123^I-5IA-85380 and corresponding rCBF patterns were reported to allow interpretation of whether the observed regional changes were receptor-specific or potentially perfusion-driven. Although a subset of DLB participants (n = 4) were receiving ChEIs at the time of scanning, the majority (n = 11) were medication-naïve. To directly address this potential confound, we conducted a post hoc analysis comparing patients on versus off cholinesterase inhibitor treatment. Although we could derive a nAChR covariance pattern distinguishing DLB patients ‘on’ (*n* = 4) versus ‘off’ (*n* = 11) AChEI medication (F₁,₁₃ = 13.6, *p* = 0.003), bootstrap resampling revealed no individual voxels significantly contributed to this pattern with high confidence (|Z|≤ 1.64, *p* > 0.05), indicating an absence of regionally specific effects. This suggests that, while the method will always produce a group-separating pattern, the result here lacks anatomical and neurobiological specificity. Nonetheless, this remains a potential confound that should be explored in larger cohorts. Although most in vivo studies using 123I-5IA-85380 have been conducted by our group, the tracer has demonstrated high selectivity and favourable kinetic properties for α4β2 nAChRs in independent validation studies [5]. Broader adoption has likely been constrained by challenges in radiochemistry, regulatory requirements, and the short shelf-life of the tracer, which limits widespread clinical availability. These factors should be considered when evaluating the generalisability of findings and designing future multicentre studies.

This study provided novel insights into some of the pathophysiology of DLB. Our findings revealed that the cholinergic ‘disease’ and ‘cognitive impairment’ related patterns shared specific topographical features within DMN (self-referential thought/memory), executive and primary visual hubs. The disease pattern also showed decreased binding in salience and limbic systems, whereas the cognitive impairment pattern exhibited preservation/upregulation of these circuits, highlighting a possible response mechanism to the cognitive changes. Moreover, the cognitive impairment pattern appeared to extend beyond memory, attentional and executive networks to include motor and visual association hubs, suggesting a more widespread involvement of these receptors across multiple brain systems and their broader role in cognition in DLB. Future research could investigate whether these network-level changes predict treatment response, as well as identification of patterns associated with specific symptomatic improvements, which could refine therapies for DLB. Notably, while donepezil and rivastigmine have the strongest evidence base for use in DLB, galantamine is the only agent with known allosteric modulatory effects on nicotinic receptors. To date, its evaluation has been limited to open-label trials. However, given the present findings and clear impacts on multiple brain systems, further investigation of galantamine as a therapeutic candidate in DLB is warranted.

## Data Availability

The datasets generated and/or analysed during the current study are not publicly available due to the inclusion of potentially identifiable clinical data and ethical restrictions. However, anonymised data may be made available from the corresponding author on reasonable request, subject to approval by the relevant institutional ethics committee.
